# Non-invasive biophysical measurement of travelling waves in the insect inner ear

**DOI:** 10.1098/rsos.170171

**Published:** 2017-05-03

**Authors:** Fabio A. Sarria-S, Benedict D. Chivers, Carl D. Soulsbury, Fernando Montealegre-Z

**Affiliations:** School of Life Sciences, Joseph Banks Laboratories, University of Lincoln, Lincoln LN6 7DL, UK

**Keywords:** travelling wave, cochlea, tonotopy, hearing, laser vibrometry, katydid

## Abstract

Frequency analysis in the mammalian cochlea depends on the propagation of frequency information in the form of a travelling wave (TW) across tonotopically arranged auditory sensilla. TWs have been directly observed in the basilar papilla of birds and the ears of bush-crickets (Insecta: Orthoptera) and have also been indirectly inferred in the hearing organs of some reptiles and frogs. Existing experimental approaches to measure TW function in tetrapods and bush-crickets are inherently invasive, compromising the fine-scale mechanics of each system. Located in the forelegs, the bush-cricket ear exhibits outer, middle and inner components; the inner ear containing tonotopically arranged auditory sensilla within a fluid-filled cavity, and externally protected by the leg cuticle. Here, we report bush-crickets with transparent ear cuticles as potential model species for direct, non-invasive measuring of TWs and tonotopy. Using laser Doppler vibrometry and spectroscopy, we show that increased transmittance of light through the ear cuticle allows for effective non-invasive measurements of TWs and frequency mapping. More transparent cuticles allow several properties of TWs to be precisely recovered and measured *in vivo* from intact specimens. Our approach provides an innovative, non-invasive alternative to measure the natural motion of the sensilla-bearing surface embedded in the intact inner ear fluid.

## 1. Introduction

Among vertebrates, mammals and birds exhibit an elaborate hearing system, in which auditory perception relies on mechanical and neurophysiological processes occurring in the inner ear [1]. Frequency discrimination occurs in the cochlea, a coiled, fluid-filled structure of bone located inside the skull. Sound is decomposed in a spatial frequency map characterized as tonotopy. This is supported by an oscillatory motion travelling along the length of the basilar membrane (BM), a structure inside the cochlea, which bears the stereocilia (sensory cells). This travelling wave (TW) propagates inside the cochlea and generates an amplitude maxima response at frequency-dependent locations [2]. The mechanical displacement at resonant points stimulates the sensory receptor cells initiating a neural response.

First used to describe the motion of the BM in the cochleae of human cadavers [3], passive TWs are viewed today as the substratum for active cochlear amplification in mammals [1,4]. Phenomena analogous to TWs have been directly observed in the basilar papilla of birds (Aves) [5] and the ears of bush-crickets (Insecta) [6,7], and have also been inferred, via the timing of responses of auditory-nerve fibres, in the hearing organs of some reptiles and frogs [8,9]. In vertebrates, the structure and location of the inner ear make it almost impossible to access without altering its integrity [1,7,10]. Measurements *in vivo* have only been done through small openings in the *scala tympani* or other isolated places [10–12]. Indirectly, the spatial frequency response on the BM has also been inferred through computational models, or estimated from auditory afferent nerve fibres at selected points [13,14]. Hitherto, there lacks an easy, non-invasive approach to directly access the complex auditory processes occurring within the cochlea.

**Figure 1.**
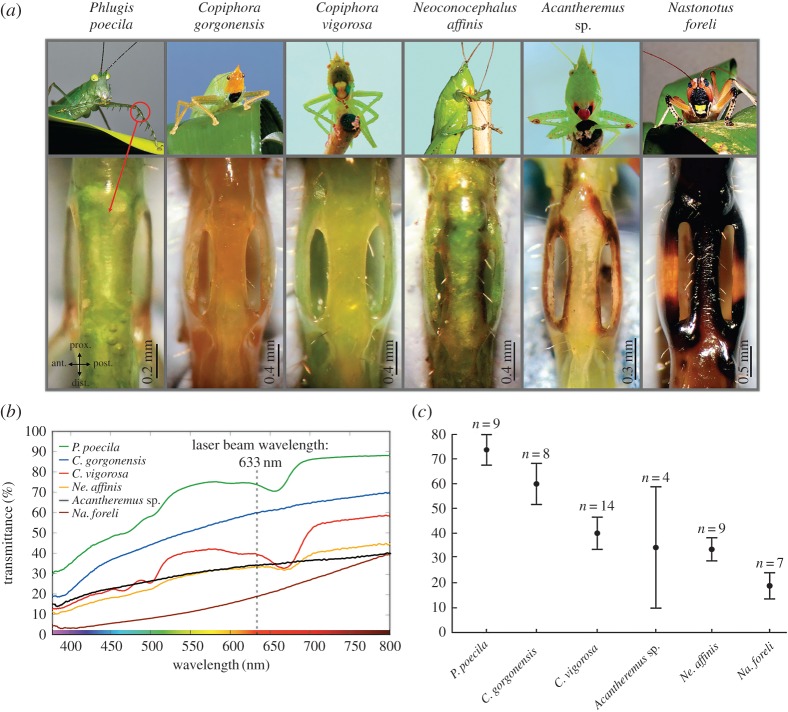
Study species and cuticle transmittance. (*a*) Species of bush-cricket (Tettigoniidae) used for the transmittance measurements. Top row: habitus of the species; bottom row: close-up view of the ear region showing the colour and level of cuticle pigmentation for each species. Red circle indicates the position of the ear in *Phlugis poecila*. (*b*) Cuticle transmittance values for all species studied. Transmittance curves (percentage of light diffused through the ear dorsal cuticle (see also figure 2*a*)) measured in the visible light spectrum (370–800 nm). (*c*) Mean transmittance values (±s.e.) of the ear dorsal cuticle of all species at the laser beam wavelength (633 nm).

Bush-crickets (Orthoptera: Tettigoniidae) are insects that exploit acoustic signals to interact with their conspecifics [15–17]. Both males and females detect sound using paired tympanal organs located on their forelegs (figure 1*a*), just below the femoro-tibial joint [18–20]. The tympanal organ is backed by an acoustic tracheal tube connecting the ear with the thoracic spiracle [21]. Just at the tympanal region, the trachea splits in two forming a fold with a triangular and slightly curved/convex surface, which contains a collection of mechanoreceptors aligned in a row forming a crest, known as the *crista acustica* (CA). Bush-crickets exhibit a highly sophisticated hearing system that includes an outer, middle and an inner ear, which exhibit basic auditory processes analogous to the mammalian system [6]. Although a large number of questions remain to be answered before the two ears can be seen as equivalent, both systems can be compared in a broad sense. The bush-cricket inner ear, formed by the CA and auditory vesicle (AV), allows effective frequency discrimination through tonotopy and TWs [22–24]. Similar to the mammalian BM in the cochlea, sound-induced TWs originate at the narrow, distal, high-frequency end of the CA, and propagate towards the wide, low-frequency, proximal region of the same structure [6,7]. This mechanical motion enhances the tonotopic response at a specific resonant location where the TW reaches its maximum displacement [25].

Innovative approaches and organisms with easy-to-access inner ears could provide alternative solutions to advance our understanding of complex auditory processes. Bush-crickets provide an ideal model, having ears which lay beneath the leg cuticle allowing researchers to measure TWs and tonotopy by removing the leg cuticle and exposing the CA [7,26]. This method has been used with electrophysiology to measure the responses of sensory cells to sound-induced mechanical forces [25]. Yet, this protocol might have negative effects on the natural operation of the ear. For example, draining the AV's fluid compromises the hydrostatic equilibrium of the system [6,27]. Alternatively, some auditory processes in the inner ear of bush-crickets have been measured non-invasively using laser Doppler vibrometry (LDV) [6]. The authors speculated that this is possible perhaps due to either translucent or thin ear cuticles, yet the mechanism by which this was possible is not understood [27]. Thus, understanding the properties of the ear cuticle is of fundamental importance for furthering research on measuring auditory activity using non-invasive techniques.

In this study, we quantified cuticle transparency across six species with different levels of cuticular pigmentation, and established the relationship between transparency, cuticle thickness and LDV measurements of auditory activity. We hypothesize that transparency is the main cuticle property allowing the precise recording and measurement of TWs and tonotopy in the inner ear of bush-crickets. Using the species with the highest cuticular transparency, the glass bush-cricket *Phlugis poecila*, we exemplify the retrieval of these complex auditory parameters from the inner ear, achieved non-invasively *in vivo*.

## 2. Material and methods

### 2.1. Specimens

Female and male adults of *Copiphora gorgonensis*, *C. vigorosa*, *P. poecila*, *Neoconocephalus affinis*, *Nastonotus foreli* and *Acantheremus* sp. were taken from colonies reared at the University of Lincoln, UK. Parental specimens were initially collected from two locations in the Colombian rainforest during December 2014 and November 2015. Collecting events took place at night (18.00–24.00) along established trails in the sampling areas, with a total of 48 h of sampling activity. The sampling locations were El palmar de la Vizcaína and the National Natural Park, Gorgona. The former is an oil palm research centre surrounded by patches of tropical rainforest situated in the valley of the Magdalena river, 32 km from the municipality of Barrancabermeja, Santander (6°59′02.3^″^ N; 73°42′20.2^″^ W). The latter is an island situated at 35 km from the Pacific coast of Colombia (2°47′ to 3°6′ N; 78°6′ to 78°18′ W). The park's ecosystem is classified as tropical wet forest with an area of 13.33 km^2^. Collected specimens were transported to the University of Lincoln, UK, under collection and exportation permit No. COR 5494-14 (issued by the Administrative Unit of National Natural Parks of Colombia).

### 2.2. Cuticle transparency measurements

Cuticle transparency was quantified by measuring the transmittance (ratio of the transmitted radiant flux to the incident radiant flux) of the cuticle covering the hearing organ. Cuticle samples were dissected from live specimens and placed in a cavity well microscope slide containing insect saline solution [28]. A 50 µm diameter optic fibre connected to a spectrophotometer (USB2000 Fibre Optic Spectrometer, Ocean Optics Inc., Oxford, UK) was placed on the projector lens in the camera ocular of a compound light microscope. For all the measurements, a 40× objective lens was used and the reference light was the illumination system of the microscope (halogen lamp), with brightness maintained at 5 V consistently for all experiments. The spectrophotometer detector unit was connected to a computer via a USB port and the collected measurements were transformed into digital format using the OOIBase32 spectrophotometer operating software (Ocean Optics Inc.). The software calculates the percentage of energy passing through a sample relative to the amount that passes through the reference:

2.1 $$\%T_{\lambda }=\frac{S_{\lambda }-D_{\lambda }}{R_{\lambda }-D_{\lambda }}\times 100\%,$$

where %*T_λ_* is the percentage of transmittance at wavelength *λ*, *S_λ_* the sample intensity, *D_λ_* the dark intensity, and *R_λ_* the reference intensity [29].

For each transmittance measurement, a reference spectrum was taken with the light source on and a blank in the sampling region. The dark reference spectrum was taken with the light path blocked, and a stray light correction was applied using boxcar pixel smoothing and signal averaging (10 averages).

### 2.3. Artificial actuator vibrations measured through transparent cuticle

**Figure 2.**
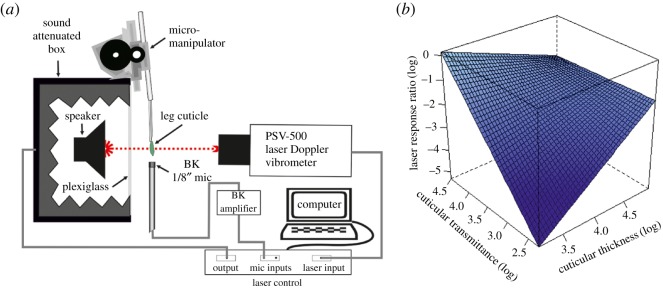
Effect of cuticle transmission and thickness on LDV experiments. (*a*) Diagram of experimental protocol for obtaining laser response ratios from freshly dissected ear cuticle. See text for details. Image not to scale. (*b*) Relationship of cuticle transmittance, cuticle thickness and laser response ratio.

A piece of freshly dissected cuticle from the dorsal ear area and a reference vibratory surface were used to evaluate the effects of the cuticle transparency on the LDV measurements, and to investigate whether the laser records ear vibrations on the cuticle, or on the CA through the cuticle. Ear top cuticles were dissected from one of the forelegs of live specimens from all species excluding *Ne. affinis* and fixed with a mixture of beeswax (Fisher Scientific, Bishop Meadow Road, Loughborough, UK) and colophonium (Sigma-Aldrich, Dorset, UK) to the tip of a copper rod (0.632 cm diameter and 23 cm long). Using a micromanipulator, the external surface of the sample was placed perpendicular between the laser head and the cone of a tweeter speaker enclosed in a custom-made acoustic attenuating box (figure 2*a*). A 30 kHz pure tone was used as a reference signal and a 1/8^″^ condenser microphone (Brüel & Kjaer, 4138-A-015 and preamplifier model 2670, Brüel & Kjaer, Nærum, Denmark) was positioned approximately 2–3 mm from the cuticle to monitor the acoustic isolation of the attenuating box and to ensure that the sound stimulus was not eliciting vibrations on the cuticle. The laser beam was focused on the cuticle and a digital scanning grid of approximately 450 points was set on the dorsal surface of the piece of cuticle. The recording time for each of the measuring points was 32 ms (5 averages), with a sampling rate of 512 kHz. The vibratory response was measured in displacement after applying a 1 kHz high-pass filter. As a control, the cuticle was removed and the surface of the speaker was scanned using the same settings and grid of points. The effect of the cuticle on the laser signal was estimated by calculating the ratio between the displacement response of the laser beam through the cuticle and onto the vibrating surface, and the control displacement response (the beam directly onto the vibrating surface with no cuticle). In this way, higher laser displacement response ratios indicate that less laser signal is being blocked, or otherwise affected, by the beam travelling through the cuticle. Lower laser displacement response ratios indicate greater levels of the laser signal is being affected by the cuticle.

### 2.4. Cuticle thickness

Cuticle thickness was measured to evaluate the effects of this property on the laser signal response. For this, the previously dissected cuticle samples were cut transversally lengthwise down the midpoint of the sample. Samples were then placed on an aluminium scanning electron microscope (SEM) stub using carbon tape. Digital images were captured and analysed with an FEI Inspect S50 microscope (FEI, Hillsboro, OR, USA). Measurements were made with the graphics software Coreldraw X7 (Corel Corporation, Ottawa, Canada) using the dimension tool and adjusting the scale to real-world values using the scale bar from each individual SEM image (electronic supplementary material, figure S1).

### 2.5. Mounting the specimens for laser Doppler vibrometry measurements of travelling waves

Protocols for measuring ear activity with LDV follow Montealegre-Z *et al*. [6]. For the LDV experiments, insects were initially anaesthetized with a triethylamine-based mix (FlyNap®, Carolina Biological Supply Company, Burlington, NC, USA) to facilitate the fastening to a horizontal brass platform (5 mm wide, 1 mm thick and 70 mm long). The dorsal pronotal area and legs, except for the frontal pair, were fixed to the platform using a mixture of beeswax and colophonium. The front legs were restrained using brass wires, which allowed positioning of the tibia and femur in a 90° angle. Additionally, the brass plate was attached to an articulated aluminium rod (150 mm long, 8 mm diameter) allowing the dorsal surface of the ear to be placed perpendicular to the scanner's laser beam. All experiments were carried out inside an acoustic booth (IAC Acoustics, Series 120a, internal length 2.40 m, width 1.8 m and height 1.98 m), at room temperature (24–26°C) and relative humidity of 32–35%. The acoustic booth provides an internal reduction to external noise of at least 59 dB at 2 kHz and above (manufacturers information). The scanning head of the laser and the experimental set-up were placed on a vibration isolation table (Optical Table Breadboard, Pneumatic Vibration Isolation (1 m × 1 m area), Melles Griot, Rochester, NY, USA).

### 2.6. Laser Doppler vibrometry measurements of travelling waves

The sound-induced vibration pattern of the ear was measured using a micro-scanning laser Doppler vibrometer (Polytec PSV-500; Waldbronn, Germany) fitted with a close-up attachment. The mounted specimens were positioned so that the cuticle overlaying the ear was perpendicular to the lens of the laser unit. A loudspeaker was positioned 30 cm ipsilateral to the specimen to broadcast the sound stimulus (electronic supplementary material, figure S2). Periodic chirps were used as the acoustic stimulus, generated by the Polytec software (PSV 9.2), passed to an amplifier (A-400, Pioneer, Kawasaki, Japan), and sent to the loudspeaker (Ultrasonic Dynamic Speaker Vifa, Avisoft Bioacoustics, Glienicke, Germany). The periodic chirps contained frequencies between 5 and 80 kHz, and the stimulus was flattened, so all frequencies were represented at 60 ± 1.5 dB (SPL re 20 µPa) at the position of the ear. A 1/8^″^ microphone (Brüel & Kjaer, 4138-A-015 and preamplifier model 2670, Brüel & Kjaer, Nærum, Denmark) was placed to monitor and record the acoustic stimulus at the position of the ear as a reference (electronic supplementary material, figure S2). The laser system was used in scan mode. A grid of scan points on the dorsal surface of the CA was established using the PSV 9.2 acquisition software (Polytec, Waldbronn, Germany). Depending on the size of the insect's leg, the actual number of measuring points per grid varied among specimens, with approximately 800 points per ear. Within the frequency domain setting of the vibrometer, a frequency spectrum was calculated for each point using an FFT with a rectangular window, at a sampling rate of 256 000 samples s^−1^, 64 ms sampling time with a frequency resolution of 15.625 Hz. A high-pass filter of 1 kHz was applied to both the vibrometer and reference microphone signals during the scanning process.

### 2.7. Data analysis

The relationship between laser response (a ratio), cuticular thickness (µm) and cuticular transmittance (%) was analysed using linear mixed effects (LMMs). Species was fitted as a random effect to account for species-differences in samples sizes. Parameters were logged before analysis. Models with and without interaction terms between cuticular thickness and cuticular transmittance were tested using likelihood ratio tests. The inclusion of the interaction significantly improved the model ($\chi_{1}^{2}=8.54$, *p* < 0.001). The relationship between cuticular thickness and transmittance was tested with a Pearson's correlation.

Data from all scanned points were examined using the PSV 9.2 presentation software (Polytec, Waldbronn, Germany). Frequency spectra, ear displacement animations and oscillation profiles were produced for selected frequencies within the recorded range. Frequency spectra of the vibrometry data were normalized to those of the reference signal by computing the transfer function of the two [30]. For the TWs analysis, coordinates and displacement values from points corresponding to a 1 mm profile line set distal to proximal on the measured grid were exported as an ASCII file. The obtained data points were analysed using a custom Matlab code (Matworks Inc., Nauticks, USA), which generates plots of the TWs recorded from the scanned ears. The plots allowed us to visualize and measure the velocity response of each point in the frequency domain. The graphical representation was used to evaluate two of the TWs' criteria: asymmetric envelope and phase lag [30]. Furthermore, TWs' propagation velocity and wavelength were calculated from the phase response using the equations:

2.2 $$\begin{array}{rl} \delta_{t} & =\frac{\delta_{\phi }}{2\pi f}\;, \end{array}$$

2.3 $$\begin{array}{rl} V_{\mathrm{wave}} & =\frac{\delta_{x}}{\delta_{t}}, \end{array}$$

2.4 $$\begin{array}{rl} \;\text{and}\;\;\lambda & =\frac{2\pi \delta_{x}}{\delta_{\phi }}. \end{array}$$

Where *f* is the wave frequency (Hz), *δ_ϕ_* the phase difference (rad) between two points at different locations, *δ_t_* the travel time (s), *δ_x_* the distance travelled (m), *V*_wave_ the wave velocity and *λ* the wavelength [1,30]. We then tested the relationship between these parameters and frequency using LMMs. In each model, individual bush-cricket was fitted as a random effect. For all LMMs, degrees of freedom were calculated using Sattherwaite's approximation. Statistical analysis was carried out using the lme4 package [31] run in R v. 3.3.1 [32]

## 3. Results

### 3.1. Cuticle transmittance

We quantified cuticle transparency across six species (figure 1*a*), and established the relationship between this property, cuticle thickness and LDV measurements of auditory activity. Using a spectrophotometer, cuticle transparency was quantified by measuring the transmittance (ratio of the transmitted radiant flux to the incident radiant flux) of the cuticle covering the hearing organ. Transmittance percentage values for all measured cuticles increased with wavelength in the visible light spectrum, 370–800 nm (figure 1*b*). At the light spectrum wavelength of the LDV beam (633 nm, Polytec PSV-500; Waldbronn, Germany), the curves can be distinguished into two groups. One group with transmission values relatively high, *P. poecila* and *C. gorgonensis* with averages of 73.73% ± 3.10 and 59.93% ± 4.15, respectively (mean ± s.e., figure 1*c*). The second group includes values below 50% and it is formed by *C. vigorosa*, *Acantheremus* sp. *Ne. affinis* and *Na. foreli* with transmission percentages of 40.00% ± 3.24, 34.14 ± 12.24, 33.46% ± 2.32 and 18.82% ± 2.64, respectively (mean ± s.e.).

### 3.2. Laser Doppler vibrometry ratio response

The effect of cuticle transparency specifically in relation to transmission of light from an LDV was calculated as a ratio of the LDV response (measured as displacement) from a reference vibrating surface (a membrane on a speaker playing a sine wave, figure 2*a*), and the same surface as measured through a sample of the ear cuticle. The relationship between this LDV response and cuticle transmission, including cuticle thickness, was quantified through linear regression of these variables. Cuticle thickness was obtained by measuring cross sections of the dissected ear cuticle (electronic supplementary material, figure S1). An LMM model found that laser response ratio was significantly related to the interaction between cuticle thickness and transmittance values (LMM: cuticular thickness × transmittance *β* ± s.e. = 0.90 ± 0.31, *F*_1,18.07_ = 8.53, *p* = 0.009; LMM: cuticular thickness *β* ± s.e. = −3.52 ± 1.11, *F*_1,16.13_ = 9.96, *p* = 0.006; LMM: transmittance *β* ± s.e. = 4.08 ± 1.30, *F*_1,18.07_ = 9.82, *p* = 0.006). Lowest laser response ratio occurred when both the cuticle were thin and when transmittance was low (figure 2*b*); the highest laser response ratio occurred when transmittance was high and cuticles were thinnest (*P. poecila:* mean ± s.e. = −0.24 ± 0.07). Transmittance and cuticle thickness were not correlated (*r_p_* = −0.09, *p* = 0.667).

### 3.3. *In vivo* measurement of travelling waves

**Figure 3.**
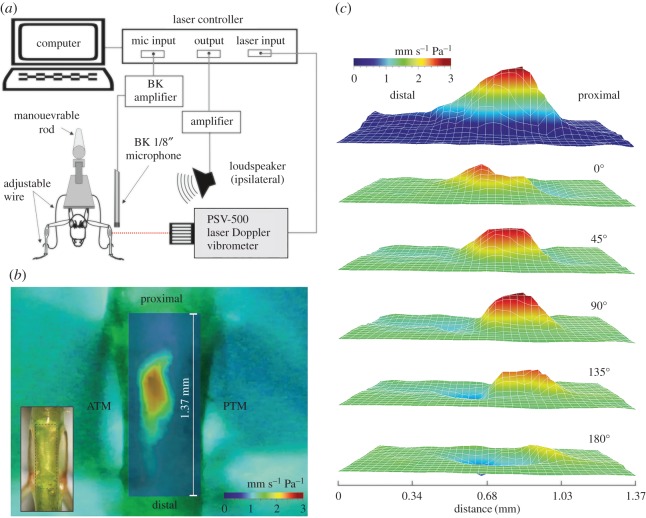
LDV experimental set-up and output. (*a*) Diagram of experimental protocol for non-invasive measurements of auditory function in bush-crickets using LDV. See text for details. Image not to scale. (*b*) Laser vibration map showing the distribution of areas of high vibration amplitude. Inset: ear area scanned during the LDV experiments. (*c*) Three-dimensional representation of the same data in *b* of a TW at 10 kHz through phases of 45° of the oscillation cycle.

**Figure 4.**
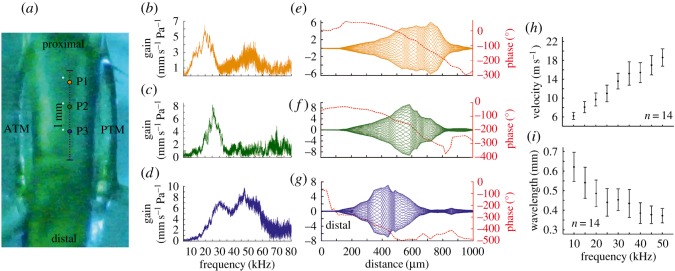
Spatial frequency mapping and TWs in the inner ear of the glass bush-cricket *P. poecila*. (*a*) Close-up view of the left leg ear showing a three-point longitudinal transect between the anterior (ATM) and posterior tympanal membrane (PTM). The locations where the maximum velocity was recorded in the ear for 19, 25 and 47 kHz are represented by P1, P2 and P3, respectively. (*b–d*) Frequency response measured as velocity gain at locations P1–P3. (*e–g*) Envelope reconstruction along the transect in *a* for 19, 25 and 47 kHz. The deflection envelopes are constructed by displaying phase increments of 10° in the full oscillation cycle. The red broken line represents the phase lag in degrees (red scale on the right) for the same frequencies and distance. The velocity (*h*) and wavelength (*i*) of the travelling wave for various frequencies in *P. poecila*.

In order to corroborate the feasibility of transparent species for *in vivo* hearing experiments, the auditory activity of specimens of *P. poecila* was investigated, as this species presented the highest transmittance values and thinnest cuticles. Non-invasive measurements of tonotopy and TWs *in vivo* were done by directly measuring the sound-induced vibration pattern of the ear using LDV (figure 3*a,* example of LDV output, figure 3*b,c*; see also electronic supplementary material, movie S1). A spatially discrete response was observed for frequencies between 10 and 60 kHz from non-invasive measurements along the length of the hearing organ (figure 4*a–d*). With increasing stimulus frequency, the maximum response shifts towards the distal part of the leg (figure 4*a*–*d*) as predicted by the TW model of cochlea function.

The measured response in the inner ear satisfies two criteria for the inference of TWs: (i) asymmetric envelope and (ii) phase lag [1]. The magnitude of CA displacement shows an asymmetric envelope around the point of maximal deflection. This point is also the location where the wave is seen to compress before dying off. TW asymmetry was evaluated as the response gain (mm s^−1^ Pa^−1^) along a transect line across the CA for different frequencies (figure 4*e*–*g*) and it was observed that the position of the maximum displacement of the TW envelope varies with frequency. At 19 kHz, the wave is asymmetrical about 720 µm along the transect (figure 4*e*), at 25 kHz, the asymmetry occurs around 577 µm (figure 4*f*) and for 47 kHz, the same phenomenon is observed approximately at 447 µm (figure 4*g*). Similarly, the phase response across the CA displays an increasing lag along the transect (figure 4*e*–*g*). The lag increases as a function of frequency; for instance, at 19 kHz, the phase lag is 281°, while at 47 kHz, the lag reaches 419° difference between the initial and final phase angle.

Velocity and wavelength of propagation are parameters of TW that can be acutely characterized with our approach. The velocity of the TW in the inner ear of *P. poecila* increased from 6.22 ± 1.22 to 18.55 ± 3.04 m s^−1^ in a frequency range of 10–50 kHz. The wavelength on the other hand decreased from 0.62 ± 0.12 to 0.37 ± 0.06 mm for the same frequency range. In our measurements, TW velocity was significantly positively related to sound frequency (LMM: *β* ± s.e. = 0.31 ± 0.02, *F*_1,103_ = 315.60, *p* < 0.001; figure 4*h*). Conversely, there was a significant decrease in wavelength size as frequency increased (LMM: *β* ± s.e. = −0.006 ± 0.001, *F*_1,103_ = 77.48, *p* < 0.001; figure 4*i*).

## 4. Discussion

We have confirmed cuticle transparency and cuticular thickness as primary factors allowing the non-invasive measurement of TWs and auditory mechanics in the bush-cricket inner ear. Furthermore, our analysis reveals that transmittance of light through the cuticle is a reliable indicator of a species' suitability for experiments specifically using LDV. The lack of correlation between cuticle transmittance and thickness indicates that pigmentation affects transparency and, in turn, laser measurements. This explains why established model species in insect hearing research like *Mecopoda elongata* [7] were not suitable in attempts of non-invasive laser measurements [27].

From the six species studied, *P. poecila* is a good model for auditory research due to its exceptional cuticle transparency and hearing capabilities. This could also apply to many species of the same subfamily (Meconematinae) within the genus *Phlugis* or related genera, which are also known as ‘glass’ or ‘crystal bush-crickets’ (or katydids). Male *P. poecila* produce calling songs to attract females using a broadband call with a main carrier frequency peaking around 50 kHz (electronic supplementary material, figure S4). Our non-invasive approach shows that the ears of this species also incorporate a wide spectrum of frequencies from the audible to the ultrasonic range (at least 6–70 kHz, figure 4), and overlap the hearing ranges of humans and other vertebrates.

Several parameters of the auditory process could be measured non-invasively from the inner ear using LDV. Yet, to which extent some of the values recovered are real is unknown. Scattering of the laser beam at the cuticle (externally and internally) and at the AV might have an effect on the final values measured (for instance, mechanical amplification). The presence of a liquid medium between the cuticle and the CA reduces the laser beam scattering by providing a refractive index-matching effect [33]. The chemical composition of the AV fluid remains unknown, but it is likely that its refractive index, as reported for the haemolymph of other insects [34], is higher than that of water (1.33). Therefore, due to a possible high refractive index, the AV fluid might increase the resolving power between the cuticle and the CA, as occurs with the use of immersion oils in light microscopy [35]. Finally, we think that the AV's geometry combined with the refractive index of the liquid together have an optical effect analogous to a plano-convex lens. As a consequence, this property increases the numerical aperture of the laser beam while reducing the characteristic irradiance loss of a Gaussian beam [36]. While the refractive index of the AV fluid was not measured in this study, future efforts should aim to account for this optical effect and to correct the LDV values of velocity/displacement accordingly [37,38].

Taking advantage of the high level of cuticle transparency and wide-frequency bandwidth of auditory perception (electronic supplementary material, figure S3) in *Phlugis* spp., we corroborated the use of bush-crickets as an alternative system for the non-invasive study of auditory processes. The observed phase lag and asymmetric envelope along the CA (figure 4*e–g*) allowed us to characterize the auditory response as a TW with displacement maxima at tonotopically specific locations. The obtained TW velocities and wavelengths are shown (figure 4*h*,*i*). These parameters have been calculated in the bush-cricket *M. elongata* by opening the cuticle and draining the natural AV fluid [12]. The data presented here were collected non-invasively from an intact system, reducing the effects of surgically opening the inner ear cavity (e.g. changes in the hydrostatic pressures and fluid density) [6,27]. It has been shown that the amplitude velocity of the CA decreases rapidly when the system is altered by, for example, draining its fluid, and that this operation also alters the phase of the tympanal vibration [27]. However, the decrease in TW wavelength with increasing frequency, and the corresponding increase in TW velocity, presented here, are in good agreement with predictions of TW function as observed in vertebrate [1,39] and invertebrate [6,7] models.

Understanding hearing processes such as tonotopy and TWs in mammals is crucial to further auditory research regarding nonlinear processes within the cochlea [13]. As mentioned before, anatomical limitations for accessing and obtaining data *in vivo*, and in an intact system, have been a major drawback in this field. Recently, methods for the measurement of auditory activity *in vivo* have improved notably for mammals. Developments with various techniques using optical coherence tomography (OCT) provide a visual technique for depth-resolved displacement measurements of TWs through the bony shell that protects the cochlea [12,40]. Although such OCT techniques appear to be non-invasive, it still requires the middle ear bulla to be surgically treated to allow visual access to the cochlea. This highlights the importance of developing novel and non-invasive techniques for the acquisition of TW data, as an important part of the complex auditory system. Attempts to relate the biomechanical tonotopy to the tuning of the corresponding sensory cells in bush-crickets have produced important advances in this field [23], and the methodology presented here provides an opportunity for refinement of currently accepted experimental protocols. While research in bush-crickets' hearing has advanced in the last four decades, fine-scale details of certain auditory mechanics remain unknown. For example, the reduced number of auditory sensory neurons, and the short length of the CA, suggests that the bush-cricket ear has poor frequency resolution [7,30,41]. To resolve these and future questions about such micro-scale ears, efforts should focus on robust and rigorous model systems, otherwise these phenomena will remain elusive.

## 5. Conclusion

The transparent cuticle effectively supports the visualization and measurement of the auditory activity with no manipulation of the hearing organ required. The main advantage of this approach is that it overcomes the need for surgical intervention (i.e. removing the cuticle). Additionally, the ability to image through the cuticle allows an alternative approach to traditional electrophysiology, such as the use of voltage-sensitive dyes to follow neuron activity in real time of the mechano-sensory cells involved in the hearing process [42–44]. Furthermore, from the point of view of invasive experimental protocols, invertebrates, and especially insects, are ideal substitutes within the 3Rs framework [45]. This work achieves not only *replacement*, by providing a possible alternative to vertebrate models, but also *refinement*, by using intact systems and non-invasive measurement. As animals are unharmed during measuring, this has the potential to also *reduce* animal usage.

The bush-cricket inner ear is functionally and structurally less complex, yet smaller than those of mammals. For instance, the number of mechano-sensory cells is considerably lower in bush-crickets. Even so, the physical principles underlying hearing in mammals are the same for hearing in bush-crickets [46]. The bush-cricket frequency analyser organ (the CA-AV) is uncoiled and the tonotopic organization takes place in a relatively short distance (approximately one-third of the length of the mammalian BM), and individual cap cells are visible on the surface of the tectorial membrane along the CA (electronic supplementary material, figure S3). Such features provide unprecedented opportunity for experimental manipulation and, by the methodology presented here, for the collection of high-quality data. For example, a tentative application of such studies would be the investigation of an analogous mechanical origin of the TWs observed in the cochlea, and currently, two hypotheses have been proposed to explain this phenomenon. Firstly, that TWs arise from anisotropic properties of the BM, resulting in tonotopically arranged displacement maxima causing excitation of the sensory cells [1]. Secondly, that the observed TW is a by-product of independently resonating sensory cells, coupled by a tectorial membrane [47]. We believe that this type of study, and novel experimental designs, may open avenues of research which help answer such fundamental questions in auditory mechanics, and could provide insights into the evolution of acoustic perception, the likes of which cannot be attained by only investigating mammalian models.

## Supplementary Material

Supplementary Figures

## Data accessibility

Raw data for ear cuticle transparency (measured as transmittance), ear cuticle thickness and measurement of travelling wave parameters (wavelength and velocity) have been stored in Dryad repository (http://dx.doi.org/10.5061/dryad.m35k3) [48].

## References

[1] Robles L, Ruggero MA 2001 Mechanics of the mammalian cochlea. Physiol. Rev. 81, 1305–1352. 11427697 10.1152/physrev.2001.81.3.1305 PMC3590856

[2] Dallos P 1992 The active cochlea. J. Neurosci. 2, 4575–4585. 10.1523/JNEUROSCI.12-12-04575.1992 PMC6575778 1464757

[3] von Békésy G 1960 Experiments in hearing. New York, NY: McGraw-Hill.

[4] Hudspeth AJ 2014 Integrating the active process of hair cells with cochlear function. Nat. Rev. Neurosci. 15, 600–614. (doi:10.1038/nrn3786) 25096182 10.1038/nrn3786

[5] Gummer AW, Smolders JW, Klinke R 1987 Basilar membrane motion in the pigeon measured with the Mössbauer technique. Hear. Res. 29, 63–92. (doi:10.1016/0378-5955(87)90206-1) 3654398 10.1016/0378-5955(87)90206-1

[6] Montealegre-Z F, Jonsson T, Robson-Brown KA, Postles M, Robert D 2012 Convergent evolution between insect and mammalian audition. Science 338, 968–971. (doi:10.1126/science.1225271) 23162003 10.1126/science.1225271

[7] Palghat Udayashankar A, Kössl M, Nowotny M 2012 Tonotopically arranged traveling waves in the miniature hearing organ of bushcrickets. PLoS ONE 7, e31008 (doi:10.1371/journal.pone.0031008) 22348035 10.1371/journal.pone.0031008 PMC3278424

[8] Hillery CM, Narins PM 1984 Neurophysiological evidence for a traveling wave in the amphibian inner ear. Science 225, 1037–1039. (doi:10.1126/science.6474164) 6474164 10.1126/science.6474164

[9] Smolders JWT, Klinke R 1986 Synchronized responses of primary auditory fibre-populations in *Caiman crocodilus* (l.) to single tones and clicks. Hear. Res. 24, 89–103. (doi:10.1016/0378-5955(86)90052-3) 3771380 10.1016/0378-5955(86)90052-3

[10] Young E 2007 Physiological acoustics. In Springer handbook of acoustics (ed. Rossing TD), pp. 429–457. New York, NY: Springer.

[11] Russell I, Nilsen K 1997 The location of the cochlear amplifier: spatial representation of a single tone on the guinea pig basilar membrane. Proc. Natl Acad. Sci. USA 94, 2660–2664. (doi:10.1073/pnas.94.6.2660) 9122252 10.1073/pnas.94.6.2660 PMC20145

[12] Lee HY, Raphael PD, Park J, Ellerbee AK, Applegate BE, Oghalai JS 2015 Noninvasive *in vivo* imaging reveals differences between tectorial membrane and basilar membrane traveling waves in the mouse cochlea. Proc. Natl Acad. Sci. USA 112, 3128–3133. (doi:10.1073/pnas.1500038112/-/DCSupplemental) 25737536 10.1073/pnas.1500038112 PMC4364183

[13] Elliott SJ, Shera CA 2012 The cochlea as a smart structure. Smart Mater. Struct. 21, 064001 (doi:10.1088/0964-1726/21/6/064001) 10.1088/0964-1726/21/6/064001 PMC3494087 23148128

[14] Lagarde MMM, Drexl M, Lukashkina VA, Lukashkin AN, Russell IJ 2008 Outer hair cell somatic, not hair bundle, motility is the basis of the cochlear amplifier. Nat. Neurosci. 11, 746–748. (doi:10.1038/nn.2129) 18516034 10.1038/nn.2129

[15] Gerhardt HC, Huber F 2002 Acoustic communication in insects and anurans: common problems and diverse solutions, pp. 9–47. Chicago, IL: The University of Chicago Press.

[16] Greenfield MD 2002 Signalers and receivers: mechanisms and evolution of arthropod communication, pp. 174–218. Oxford, UK: Oxford University Press.

[17] Gwynne DT 2001 Katydids and bush-crickets: reproductive behaviour and evolution of the Tettigoniidae. Ithaca, NY: Cornell University Press.

[18] Bailey WJ 1990 The ear of the bushcricket. In The Tettigoniidae: biology, systematics and evolution (eds Bailey WJ, Rentz DCF), pp. 217–247. Bathurst, Australia: Crawford House Press.

[19] Hoy RR, Robert D 1996 Tympanal hearing in insects. Annu. Rev. Entomol. 41, 433–450. (doi:10.1146/annurev.ento.41.1.433) 15012336 10.1146/annurev.en.41.010196.002245

[20] Yack JE 2004 The structure and function of auditory chordotonal organs in insects. Microsc. Res. Tech. 63, 315–337. (doi:10.1002/jemt.20051) 15252876 10.1002/jemt.20051

[21] Jonsson T, Montealegre-Z F, Soulsbury CD, Brown KAR, Robert D 2016 Auditory mechanics in a bush-cricket: direct evidence of dual sound inputs in the pressure difference receiver. J. R. Soc. Interface 13, 20160560 (doi:10.1098/rsif.2016.0560) 27683000 10.1098/rsif.2016.0560 PMC5046957

[22] Oldfield BP 1982 Tonotopic organization of auditory receptors in Tettigoniidae (Orthoptera, Ensifera). J. Comp. Physiol. 147, 461–469. (doi:10.1007/BF00612011)

[23] Römer H 1983 Tonotopic organization of the auditory neuropile in the bushcricket *Tettigonia viridissima*. Nature 306, 60–62. (doi:10.1038/306060a0)

[24] Stolting H, Stumpner A 1998 Tonotopic organization of auditory receptors of the bushcricket *Pholidoptera griseoaptera* (Tettigoniidae, Decticinae). Cell Tissue Res. 294, 377–386. (doi:10.1007/s004410051187) 9799453 10.1007/s004410051187

[25] Hummel J, Schöneich S, Kössl M, Scherberich J, Hedwig B, Prinz S, Nowotny M 2016 Gating of acoustic transducer channels is shaped by biomechanical filter processes. J. Neurosci. 36, 2377–2382. (doi:10.1523/jneurosci.3948-15.2016) 26911686 10.1523/JNEUROSCI.3948-15.2016 PMC6705494

[26] Palghat Udayashankar A, Kössl M, Nowotny M 2014 Lateralization of travelling wave response in the hearing organ of bushcrickets. PLoS ONE 9, e86090 (doi:10.1371/journal.pone.0086090) 24465889 10.1371/journal.pone.0086090 PMC3897617

[27] Montealegre-Z F, Robert D 2015 Biomechanics of hearing in katydids. J. Comp. Physiol. A 201, 5–18. (doi:10.1007/s00359-014-0976-1) 10.1007/s00359-014-0976-1 25515594

[28] Fielden A 1960 Transmission through the last abdominal ganglion of the dragonfly nymph *Anax imperator*. J. Exp. Biol. 37, 832–844.

[29] Ocean Optics Inc. 2001 Usb2000 fiber optic spectrometer: installation and operation manual. Dunedin, FL: Ocean Optics Inc.

[30] Windmill JFC, Gopfert MC, Robert D 2005 Tympanal travelling waves in migratory locusts. J. Exp. Biol. 208, 157–168. (doi: 10.1242/jeb.01332) 15601886 10.1242/jeb.01332

[31] Bates D, Mächler M, Bolker B, Walker S 2015 Fitting linear mixed-effects models using lme4. Journal of Statistical Software 67, 1–48. (doi: 10.18637/jss.v067.i01)

[32] R Development Core Team. 2016 R: a language and environment for statistical computing. Vienna, Austria: R Foundation for Statistical Computing.

[33] Vargas G, Chan EK, Barton JK, Rylander HG, Welch AJ 1999 Use of an agent to reduce scattering in skin. Lasers Surg. Med. 24, 133–141. (doi:10.1002/(SICI)1096-9101(1999)24:2<133::AID-LSM9>3.0.CO;2-X) 10100651 10.1002/(sici)1096-9101(1999)24:2<133::aid-lsm9>3.0.co;2-x

[34] Miyajima S 1982 Refractive index in hemolymph and gut juice of the silkworm infected with some viruses. J. Sericult. Sci. Jpn 51, 176–181. (doi:10.11416/kontyushigen1930.51.176)

[35] Cargille JJ 1985 Immersion oil and the microscope. *New York Microscopical Society Yearbook*, 2nd edn. Cargille-Sacher Laboratories, Inc.

[36] Martí Duocastella CF, Serra P, Diaspro A 2015 Sub-wavelength laser nanopatterning using droplet lenses. Sci. Rep. 5, 16199 (doi:10.1038/srep16199) 26541765 10.1038/srep16199 PMC4635425

[37] Marsili R, Pizzoni L, Rossi G 2000 Vibration measurements of tools inside fluids by laser Doppler techniques: uncertainty analysis. Measurement 27, 111–120. (doi:10.1016/S0263-2241(99)00062-7)

[38] Sapozhnikov O, Morozov A, Cathignol D 2009 Acousto-optic interaction in laser vibrometry in a liquid. Acoust. Phys. 55, 365–375. (doi:10.1134/S1063771009030129)

[39] Şerbetçioğlu MB, Parker DJ 1999 Measures of cochlear travelling wave delay in humans: I. Comparison of three techniques in subjects with normal hearing. Acta Otolaryngol. 119, 537–543. (doi:10.1080/00016489950180757) 10478592 10.1080/00016489950180757

[40] Warren RL et al. 2016 Minimal basilar membrane motion in low-frequency hearing. Proc. Natl Acad. Sci. USA 113, E4304–E4310. (doi:10.1073/pnas.1606317113) 27407145 10.1073/pnas.1606317113 PMC4968750

[41] Rhode WS, Recio A 2000 Study of mechanical motions in the basal region of the chinchilla cochlea. J. Acoust. Soc. Am. 107, 3317–3332. (doi:10.1121/1.429404) 10875377 10.1121/1.429404

[42] Nikitin E, Aseev N, Balaban P 2015 Improvements in the optical recording of neuron activity using voltage-dependent dyes. Neurosci. Behav. Physiol. 45, 131–138. (doi:10.1007/s11055-015-0050-7)

[43] Baden T, Hedwig B 2010 Primary afferent depolarization and frequency processing in auditory afferents. J. Neurosci. 30, 14 862–14 869. (doi:10.1523/JNEUROSCI.2734-10.2010) 10.1523/JNEUROSCI.2734-10.2010 PMC6633615 21048145

[44] Isaacson MD, Hedwig B 2017 Electrophoresis of polar fluorescent tracers through the nerve sheath labels neuronal populations for anatomical and functional imaging. Sci. Rep. 7, 40433 (doi:10.1038/srep40433) 28084413 10.1038/srep40433 PMC5233955

[45] Guhad F 2005 Introduction to the 3Rs (refinement, reduction and replacement). J. Am. Assoc. Lab. Anim. Sci. 44, 58–59. 15812977

[46] Hoy RR 1998 Acute as a bug's ear: an informal discussion of hearing in insects. In Comparative hearing: insects, pp. 1–17. New York, NY: Springer.

[47] Bell A 2012 A resonance approach to cochlear mechanics. PLoS ONE 7, e47918 (doi:10.1371/journal.pone.0047918) 23144835 10.1371/journal.pone.0047918 PMC3493581

[48] Sarria-S FA, Chivers BD, Soulsbury CD, Montealegre-Z F 2017 Data from: Non-invasive biophysical measurement of travelling waves in the insect inner ear. Dryad Digital Repository. (http://dx.doi.org/10.5061/dryad.m35k3) 10.1098/rsos.170171 PMC5451827 28573026

