# Supplementary material for: Non-invasive biophysical measurement of travelling waves in the insect inner ear
Source: R Soc Open Sci. 2017 May 3;4(5):170171. doi: 10.1098/rsos.170171 (PMC5451827; doi:10.1098/rsos.170171)
Supplement: Supplementary Figures [file rsos170171supp1.pdf]

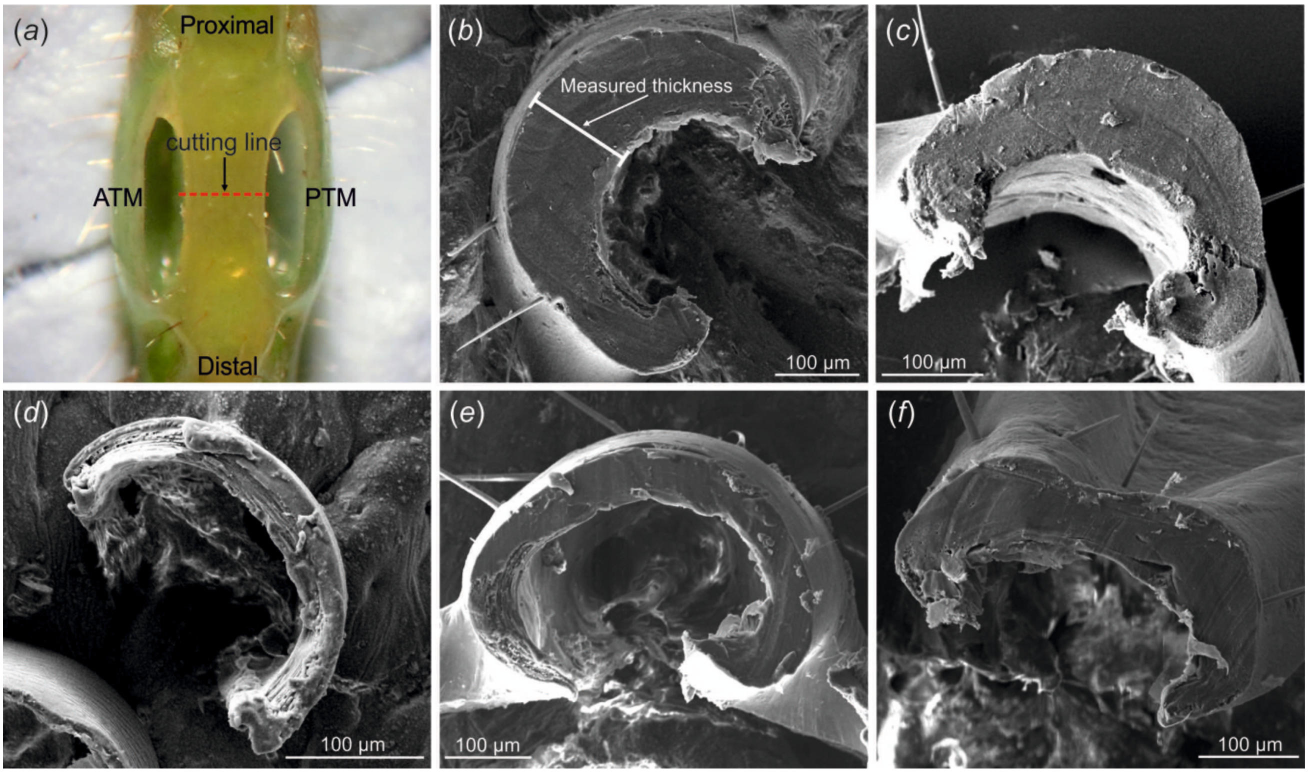

Figure S1. Examples of cuticle dissections for quantification of cuticle thickness. (*a*) Dorsal view of the ear, red line indicates location of cross section dissection. (*b*) *Copiphora vigarosa*. (*c*) *Copiphora gorgonensis*. (*d*) *Phlugis poecila*. (*e*) *Acantheremus* sp. (*f*) *Nastonotus foreli*.

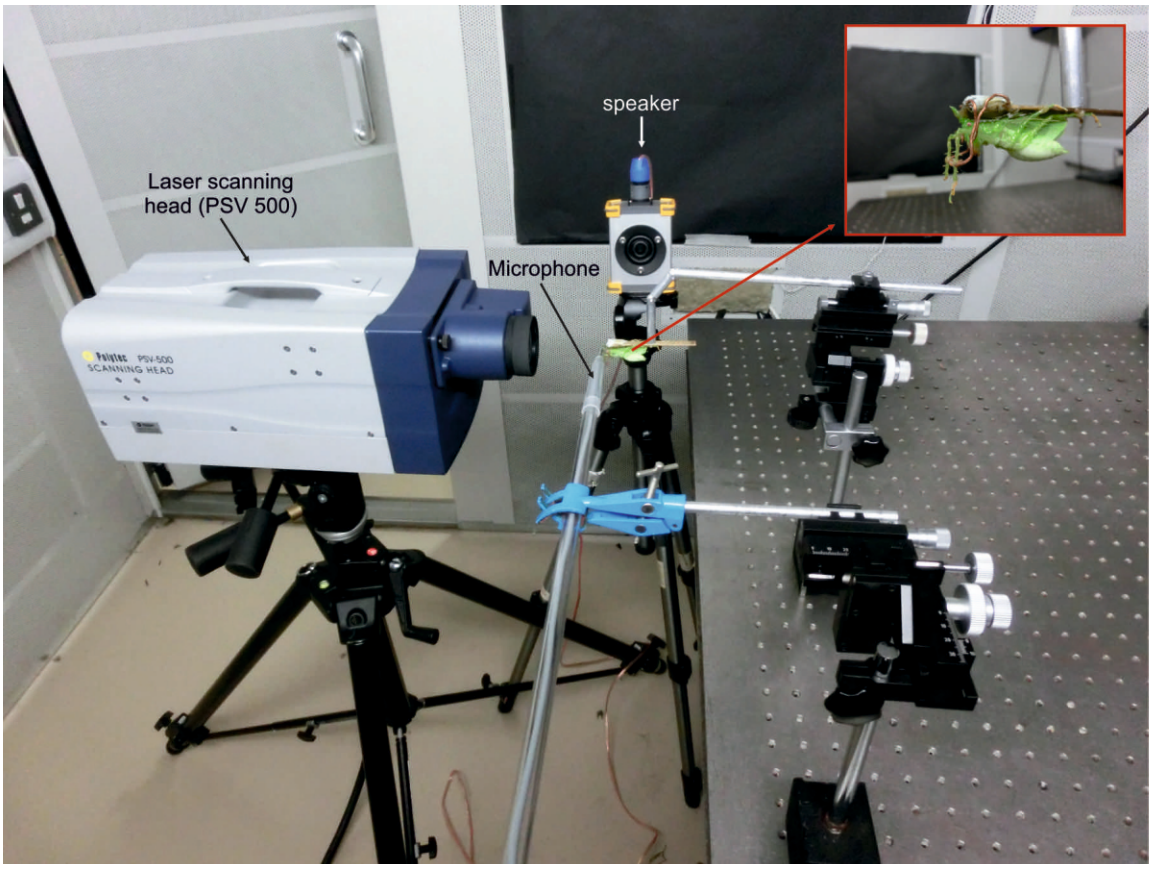

Figure S2. Experimental set-up for non-invasively measuring travelling waves in bush-crickets. See text for details. Inset: preparation of the mounted bush-cricket.

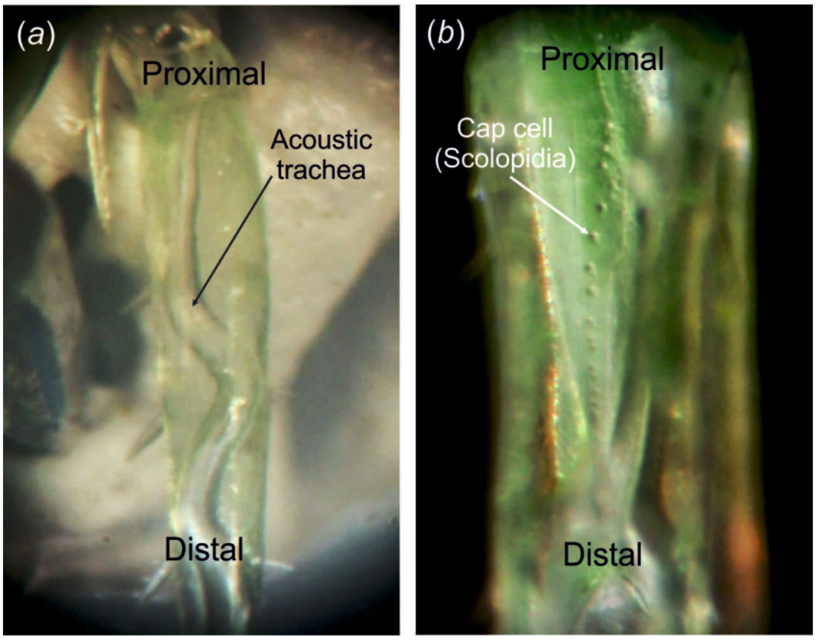

Figure S3. Cuticle transparency in a glass bush-cricket *Phlugis* sp. (*a*) Lateral view of the femur, the acoustic trachea is clearly visible through the cuticle without manipulation of the animal. (*b*) Dorsal view of the hearing organ. The cap cells (scolopidia) are visible through the cuticle.

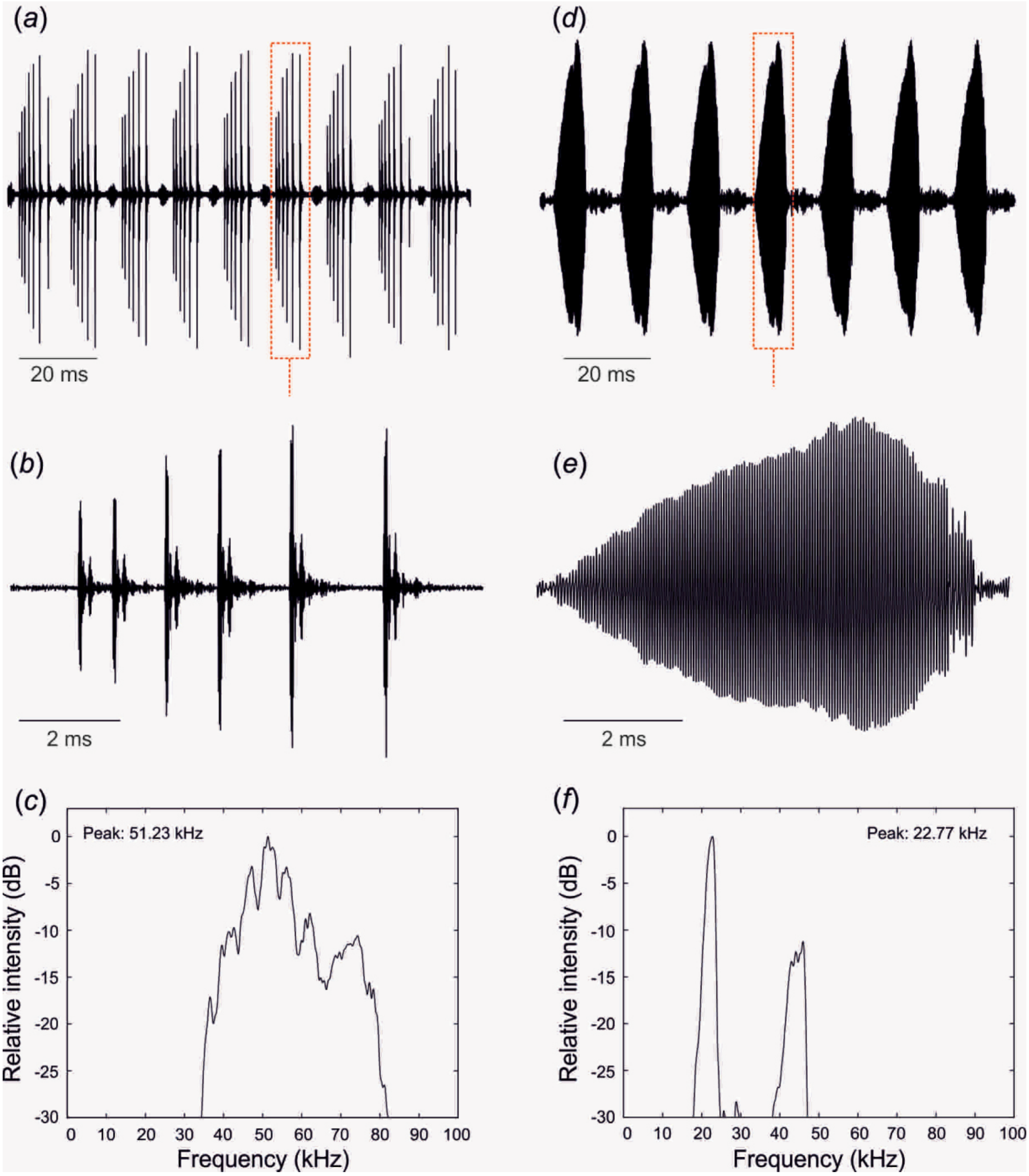

Figure S4. Acoustic analysis of the call of the two species exhibiting more cuticle light transmittance. (*a-c*) *Phlugis poecila* and (*d-f*) *Copiphora gorgonensis*. (*a*) Typical presentation of the call. (*b*) A single phonatome (closing stroke of the wings) in detail. (*c*) Spectral analysis of the phonatome in (*b*). Wide bandwidth of prevalent frequencies are apparent in the call of *P. poecila*. (*d*) Typical presentation of the call. (*e*) A single phonatome (closing stroke of the wings) in detail. (*f*) Spectral analysis of the phonatome in (*e*). Note higher tonal purity and harmonic content in the call of *C. gorgonensis*.
